# The ‘twin pandemics’? modelling and predicting the trajectories of IPV perpetration during the COVID‐19 pandemic in Australia

**DOI:** 10.1111/bjso.12911

**Published:** 2025-07-23

**Authors:** Gery C. Karantzas, Daniel A. Romano, Susan Chesterman, Emma M. Marshall, Laura Knox, Ellie R. Mullins, Nicholas Lawless, Elizabeth Ferguson, Peter G. Miller, Christopher I. Eckhardt, Pam Pilkington, Anshu Patel, Jeffry A. Simpson

**Affiliations:** ^1^ School of Psychology Deakin University Geelong Victoria Australia; ^2^ Department of Psychological Sciences Purdue University Indiana USA; ^3^ Department of Psychology University of Minneosta Minneapolis Minnesota USA

**Keywords:** COVID‐19, I^3^ model, intimate partner violence, perfect storm, social restrictions

## Abstract

Initial research suggested that intimate partner violence (IPV) increased over COVID‐19 due to social restrictions. This IPV increase during COVID‐19 has been termed the ‘twin pandemics’. Closer inspection of the evidence, however, challenges this notion. In this study, Australian residents (*N* = 608) who were either exposed to strict, prolonged lockdown orders (Victorian residents) or not (non‐Victorian residents) completed 10 waves of IPV perpetration assessment online over five months and baseline assessments of instigating factors (situational factors that increase IPV perpetration), impelling factors (personal characteristics that increase IPV perpetration) and inhibiting factors (personal and situational factors that diminish IPV perpetration). Latent profile analysis and conditional latent growth curve modelling revealed that lockdown alone did not predict IPV trajectories. However, individuals whose profiles evidenced higher instigating and impelling factors and lower inhibiting factors (i.e. perfect storm profile) demonstrated elevated physical and psychological IPV over time compared to those whose profiles evidenced lower instigating and impelling factors and higher inhibiting factors (i.e. low‐risk profile). Those with a perfect storm profile also evidenced steeper acceleration in physical and psychological IPV over time. The findings call into question the ‘twin pandemics’ notion and suggest that IPV over COVID‐19 is best predicted by a specific risk profile.

Intimate partner violence (IPV) is defined as physical, sexual or psychological harm, or the threat of such harm, within a romantic relationship. It is a serious public health issue with lifetime prevalence rates across various Western countries of approximately 27% for women and 14% for men (Office of National Statistics, 2024; World Health Organization, 2024).

Since the onset of the COVID‐19 pandemic, serious concerns have been raised about the rising rates of IPV perpetration. Initial findings indicated increases in IPV perpetration prevalence across Europe, Asia and America during the early stages of the pandemic (Das et al., 2020; Mohler et al., 2020). This phenomenon of increasing IPV perpetration rates across parts of the world that coincided with COVID‐19 has been termed the ‘twin pandemics’ or a ‘pandemic in a pandemic’ (Evans et al., 2020) and is possibly attributable to the many and varied stressors brought on by COVID‐19. These include, but are not limited to, financial stress, job insecurity, housing instability and increased household responsibilities associated with social restrictions and the pandemic‐related economic downturn. Such stressors tax individuals' ability to effectively regulate their emotions (Myruski et al., 2019) and can induce negative affect, leading to heightened anger and sometimes the enactment of IPV (Birkley & Eckhardt, 2015). Social restrictions (e.g. social distancing, lockdown orders) may also have facilitated opportunities for IPV perpetrators to commit physical and psychological violence against their partners by making it more difficult for victims to leave their homes and seek support from family, friends or domestic violence services (Kourti et al., 2023). Indeed, it has been proposed that lockdown orders may be one of the primary culprits responsible for increased rates of IPV perpetration during the pandemic (Kourti et al., 2023).

A closer inspection of all the evidence, however, suggests very mixed findings. Although some countries reported increases in IPV early in the pandemic, other countries showed no change or a decrease in IPV reports (Brink et al., 2021). In other studies, rates of violence substantially increased immediately after the implementation of a lockdown, then returned to rates comparable with pre‐pandemic levels, even though lockdown was still in effect (Ashby, 2020; Nivette et al., 2021; Payne et al., 2022). These varied findings indicate the need to develop a clearer understanding of both the rates and trajectories of IPV perpetration across the entire period of the pandemic, including during (and out of) lockdown periods. One way to do this is by conducting growth curve analysis, which can facilitate the modelling of linear and non‐linear changes. Growth curve modelling can significantly contribute to a more thorough understanding of IPV change dynamics over the COVID‐19 pandemic. Specifically, this type of modelling approach can help to identify levels or thresholds at which IPV is likely to increase and decrease across time, and whether changes in IPV are indeed associated with particular social restrictions such as lockdown orders. Trajectory analyses have already been employed to advance understanding in the psychological sciences with regards to people's trauma and distress responses over the course of COVID‐19 (e.g. Chen et al., 2022).

However, to determine whether the trajectory of IPV perpetration is associated with lockdown orders requires the implementation of a study design that not only includes participants that are assessed pre, during and after lockdown, but that compares participants that experience lockdown restrictions with participants who are not subjected to lockdown restrictions. The COVID‐19 pandemic in Australia provides a novel naturalistic study context to examine the trajectories of IPV perpetration across citizens who experienced differences in COVID‐19 social restrictions.

## Australia and COVID‐19

The Australian experience of the COVID‐19 pandemic provides a unique opportunity to study the effects of strict lockdowns on IPV (Miller et al., 2023). After the first national lockdown during the first quarter of 2020, Victoria, Australia's second most populated state, enacted a second strict lockdown that lasted from early July until late October 2020 while the rest of the country had minimal or no social restrictions.^1^ The Australian context, therefore, represents a ‘natural experiment’ allowing one to investigate how differences in lockdown status affected the trajectory of IPV over time. In the current study, we measured IPV every 2 weeks over 10 assessment waves, prior *and* post Victoria's second lockdown. This prospective design, coupled with an Australian sample that included citizens living either within or outside of Victoria, provides a good test of whether IPV in the general community was systematically associated with lockdown orders.

## Situating IPV and COVID‐19 with an integrative model

Understanding the occurrence of IPV perpetration within the context of COVID‐19, and more broadly, requires the consideration of the multitude of personal and situational factors that contribute to the perpetration of violence. Theoretical and empirical work suggests that the perpetration of IPV is predicted by several important individual and contextual risk factors (Capaldi et al., 2012). Thus, the application of integrative frameworks can provide deeper insights into the dynamic interplay among these risk factors for IPV across the COVID‐19 pandemic. One integrative framework is the I^3^ model (pronounced ‘I‐cubed’; Finkel & Eckhardt, 2013). This model proposes that the perpetration of IPV is the result of three broad but related factors: instigating, impelling and inhibiting factors. *Instigating* factors are situations and events that provide the initial momentum towards relationship aggression. *Impelling* factors are personal characteristics that increase the urge to aggress. Inhibiting factors are personal and situational factors that diminish the urge to aggress, even in the presence of provoking situations and impelling traits. Grouped within this category are also those factors that mitigate inhibition (and thus disrupt inhibitory control), with these variables referred to as (dis)inhibiting factors (Finkel, 2014). Below we outline key impelling, instigating and inhibiting factors for which there is good evidence to support their inclusion as predictors of IPV perpetration.

## Instigating factors

Research to date has identified a number of negative partner behaviours that can increase the risk of IPV perpetration. For example, being the target of a partner's destructive conflict behaviours (e.g. hostility, criticism and contempt) is associated with the perpetration of IPV (Heyman et al., 2022). Similarly, being the target of dehumanisation by a romantic partner is considered to increase the risk of perpetrating maltreatment and aggression (Karantzas, Simpson, & Haslam, 2023). Being the target of a partner's negative behaviours is considered to be internalized as disregard and invalidation that can incite aggressive retaliation (Finkel & Eckhardt, 2013).

## Impelling factors

Impelling factors are characteristics and qualities a person harbours that set the foundation for the enactment of aggression. Thus, impelling factors can be considered to reflect a person's psychological blueprint to aggress. The research on the impelling factors related to IPV perpetration has largely emerged from individual differences and developmental perspectives on IPV. From the individual differences perspective, research has found that dark personality traits such as narcissism, psychopathy, Machiavellianism and trait aggression predict IPV perpetration (e.g. Ruddle et al., 2017; Webster et al., 2014). All traits share the core features of callousness and manipulation which can manifest in an individual's cruel disregard for a partner and desire to control them (Finkel & Eckhardt, 2013). Similarly, the perpetration of dehumanisation has been found to correlate with abuse in relationships (Pizzirani & Karantzas, 2019) as it enables an individual to act abusively and violently because they perceive the target of dehumanization to be someone who is less than human and inferior, and thus not worthy of moral consideration or respectful treatment (Pizzirani & Karantzas, 2019). Research from both personality and developmental perspectives has also identified attachment insecurity (i.e. individual differences in people's distrust of close others and anxiety over abandonment) to be associated with IPV perpetration (e.g. Knox et al., 2023; Velotti et al., 2018). This is because insecurely attached people are thought to perpetrate violence in order to meet their attachment needs for emotional distance or excessive approval and closeness (Knox et al., 2023). Finally, psychological distress in the form of symptoms of depression and anxiety have been found to heighten the tendency to perpetrate IPV as people's negative cognitive‐affective states bias their perceptions of partners and also increase their difficulty to regulate their emotions which can strengthen the urge to aggress against a romantic partner (Spencer et al., 2019).

## Inhibiting (and [dis]inhibiting) factors

An array of personal and situational variables have emerged as factors that either inhibit perpetration or weaken an individual's ability to inhibit aggressive behaviour (Chester & DeWall, 2018; Eckhardt et al., 2021; Finkel & Eckhardt, 2013). The variables that are conceptualised as inhibitory and (dis)inhibitory reflect those that increase inhibitory control towards aggression or interfere with self‐regulatory processes and executive functioning, which help a person to resist the urge to aggress. We outline key inhibiting and (dis)inhibiting variables as they relate to IPV and/or COVID‐19.

### Inhibiting factors

Perceived partner support is thought to facilitate the inhibition of destructive behaviours such as the perpetration of IPV because the provision of support attends to partner needs for comfort and validation and thereby creates a relationship climate of respect and positive regard for one's partner and relationship (Eller et al., 2019). Relationship quality inhibits IPV as individuals that have high‐quality relationships characterised by trust, intimacy, satisfaction and alike. Therefore, they perceive their partner and relationship as better meeting their needs and thus are less motivated to engage in IPV as it may disrupt their relationship quality (Eller et al., 2019; Finkel & Eckhardt, 2013). Similarly, constructive communication involving compromise and solution‐focused responses to problems also mitigates the perpetration of IPV as individuals utilise positive approaches to deal with difficulties effectively rather than resorting to aggression (e.g. Gonzalez‐Mendez et al., 2018).

### (Dis)inhibiting factors

Research shows that the consumption of alcohol and illicit substances disrupt executive functioning and cognitive processes used to inhibit aggressive tendencies and impulses as well as the ability to accurately perceive threat and other social cues that drive behaviour (Eckhardt et al., 2019, 2021). The attenuation of these cognitive processes results in the outward enactment of IPV (Chester & DeWall, 2018; Parrott & Eckhardt, 2018). In this regard, substance misuse is considered to have a (dis)inhibitory function and thus classed within inhibitory factors as part of the I^3^ model (Finkel, 2014; Finkel & Eckhardt, 2013). Research has also found that various stressors (e.g. job and financial stress) that sit external to the couple predict IPV perpetration (e.g. Lohman et al., 2013; Schwab‐Reese et al., 2016). These external stressors are classed as (dis)inhibitory (rather than impelling or instigating) because they do not inherently increase the urge to aggress. Rather, these stressors are assumed to tax individuals' adaptive coping responses, cognitive and behavioural control, and can activate negative affective responses, such as the fight‐or‐flight response, which are associated with the enactment of IPV (e.g. Eckhardt et al., 2019). Within the context of the COVID‐19 pandemic, there is considerable evidence to suggest that the myriad of stressors experienced by individuals has challenged people's relationship functioning and heightened tendencies to perpetrate relationship aggression (e.g. Buttell et al., 2021).

## I^3^ and the perfect storm theory of IPV

Although each of the broad factors that comprise the I^3^ model (i.e. instigating, impelling and inhibiting/[dis]inhibiting) is considered to predict IPV perpetration, there is much evidence to suggest that IPV perpetration is best understood by the complex interplay between the variables that are classed as either instigating, impelling and inhibiting/(dis)inhibiting. For example, existing research has found that the association between alcohol misuse and IPV perpetration is moderated by a variety of factors including relationship quality, dark traits and psychopathology (see Kulak et al., 2025). Other work has found that stress moderates the association between individual difference factors such as attachment insecurity, aggressogenic traits and the perpetration of IPV (e.g. Hellmuth & McNulty, 2008; Knox et al., 2023). In line with the empirical evidence that supports the idea that IPV perpetration is a function of interacting variables, the I^3^ model states that IPV perpetration is most likely to occur when instigating and impelling factors are strong and inhibiting factors are weak (and/or [dis]inhibiting factors are elevated). This interaction among the three factors is termed the ‘perfect storm’ theory of IPV (Finkel, 2014) and has been used previously to successfully predict IPV perpetration (Blake et al., 2018; Eckhardt et al., 2021). Based on this theory, it should be possible to identify those individuals that evidence a perfect storm‐like profile, and that these individuals would evidence greater IPV perpetration over time compared to those that do not exhibit this profile.

## Study aims and hypotheses

In the current study, our aim was to better understand the occurrence of physical and psychological IPV perpetration over the pandemic by investigating the effects of social restrictions as well as through the application of the perfect storm theory of IPV. In relation to lockdown status and IPV, we made no a priori predictions given the heterogeneity of findings to date (e.g. Brink et al., 2021; Nivette et al., 2021; Payne et al., 2022). In relation to the perfect storm theory, we hypothesised that individuals who demonstrated a profile indicative of higher instigation and impellance, as well as lower inhibition (and higher [dis]inhibition), would demonstrate higher levels of physical and psychological IPV perpetration over the pandemic relative to individuals that deviated from this ‘perfect storm’ profile.

We addressed our aim in three stages. First, we used latent growth curve modelling (LGCM) to identify the trajectories of physical and psychological IPV perpetration over a five‐month period when Victoria enacted its second strict lockdown while the rest of Australia was subject to minimal COVID‐19 social restrictions. Second, we used latent profile analysis to identify subgroups of individuals who differed in their levels of impelling, instigating and inhibiting factors, all of which in combination pose greater risk for the perpetration of physical and psychological IPV. Third, we used conditional LGCM to test the extent to which different latent profiles and lockdown status (i.e. Australian citizens living in Victoria versus those who were not) predicted differences in trajectories of physical and psychological IPV perpetration across the pandemic. Study design and hypotheses were not pre‐registered.

## MATERIALS AND METHODS

### Participants

The sample consisted of 608 adults (M age = 31.60 years, SD = 9.1; 59.50% cisgender women, 37.50% cisgender men; 2.3% non‐binary; .2% did not specify their gender) from the general community residing in Australia (*n* = 391 non‐Victorian, *n* = 217 Victorian). All participants were currently in a romantic relationship (relationship length M = 7.20 years, SD = 7.90 years) with 38% married, 9% engaged, 29% cohabiting, 23% steady dating and 2% casually dating. In relation to sexual orientation, 79% identified as heterosexual, 13% as bisexual, 3% as lesbian, 3% as queer, 2% as gay and 1% preferred not to specify. In terms of cultural background, 77% identified as White/European, 15% as Asian, 2% as African, 2% as Hispanic and 1% as Indigenous (Aboriginal/Torres Strait Islander). The majority of the sample was employed (72%); however, income level varied with 22% earning in the lowest quartile (less than $41,000 AUD per annum), 22% earning in the medium lowest quartile, 40% in the medium highest quartile and 16% in the highest quartile.

### Materials and procedure

The study received approval from the University's Human Research Ethics Committee. All measures and data analysis code are available at https://osf.io/a5vkj/?view_only=6c89f94eef2041a984f173cbdf57d3a9.^2^ Participants were recruited through digital flyers distributed on social media platforms (e.g. Reddit) and through Prolific, an online platform used to source research participants. The digital flyers contained a link to the study website, which also linked participants to the Plain Language Statement (PLS) and online consent form. Participants recruited through Prolific were given a brief description of the study that included a link to the PLS and consent form. After reading the PLS and providing consent, all participants responded to a short screening questionnaire to determine whether they met the inclusion criteria for participation in the study (i.e. being over 18 years old, currently in a romantic relationship and not currently subject to criminal proceedings). Eligible participants were then directed to the baseline online survey. It contained measures of instigating factors (destructive conflict patterns and dehumanisation victimisation), impelling factors (attachment insecurity, dark triad traits, dehumanisation perpetration and psychological distress) and inhibiting factors (life stress [both COVID‐19‐related stress and non‐COVID‐related stress], substance use, partner support and relationship quality). The survey also included assessments of physical and psychological IPV. Following the completion of the baseline survey (T1), all participants were emailed surveys to assess IPV once every 2 weeks for an additional nine waves of assessment (T2–T10) across 5 months.

The baseline survey was administered to all participants 2 weeks prior to the state of Victoria entering lockdown restrictions. Therefore, all participants completed the baseline assessment when no parts of Australia were subject to lockdown orders. Subsequent assessments of IPV completed between T2 and T8 were during the period when Victorian participants were in a state of lockdown (a total period of 12 weeks from July to October, 2020). During this same 12‐week period, non‐Victorian participants were not subject to lockdown restrictions. The assessment of IPV completed at T9 and T10 was during the period when Victoria's lockdown restrictions were lifted. Therefore, the entire sample was not subject to lockdown restrictions during data collection for the final two waves of assessment.

Participants were remunerated $2 AUD per assessment wave. Participant remuneration was pro‐rated based on the waves completed, with a maximum remuneration of $20 AUD for completing all assessment waves. Overall, 64.5% of the final sample participated in at least five time points. The measures administered to assess baseline instigating, impelling and (dis)inhibiting factors as well as physical and psychological IPV assessed at all time points are described in the sections below.

### Instigating factors (measured at baseline)

#### Dehumanisation victimisation

The short victimisation version of the Dehumanisation in Romantic Relationships Scale (Pizzirani et al., 2019) was used to assess dehumanisation perpetration. The measure consists of 6 items such as ‘generally my partner treats me as if I embarrass them’. Items are rated on a 7‐point scale ranging from 1 (*never*) to 7 (*always*) and averaged with higher scores indicative of greater dehumanisation victimisation (*α* = .79).

#### Destructive conflict patterns

Shortened versions of the dominance (3 items), avoidance (2 items), separation (2 items) and submission (2 items) subscales of the Romantic Partner Conflict Scale (Zacchilli et al., 2009) were used to assess destructive conflict. All items are rated on a 5‐point scale from 0 (*strongly disagree*) to 4 (*strongly agree*). Scores were averaged across all items to create an overall destructive conflict patterns score, with higher scores indicative of greater destructive conflict (*α* = .83).

### Impelling factors (measured at baseline)

#### Psychological distress

The depression and anxiety subscales of the short version of the Depression, Anxiety, and Stress Scale (DASS‐21, Lovibond & Lovibond, 1995) were used to assess symptoms of depression and anxiety. Each symptom type is assessed using 7 items rated on a 4‐point scale from 0 (*did not apply to me at all—never*) to 3 (*applied to me very much, or most of the time—almost always*). Items on each subscale are summed, and an overall score for psychological distress is derived by summing across all subscales, with higher scores reflecting greater psychological distress (*α* = .93).

#### Attachment insecurity

Attachment insecurity towards a romantic partner was measured using the Experiences in Close Relationships Scale—Short Form (ECR‐S, Wei et al., 2007). The measure consists of two subscales assessing the two primary dimensions that underpin attachment insecurity: attachment anxiety (6 items; *α* = .90) and attachment avoidance (6 items; *α* = .86). Items are rated on a 7‐point scale from 1 (*strongly disagree*) to 7 (*strongly agree*) with higher scores on each subscale reflecting higher attachment insecurity.

#### Dark triad

The Dirty Dozen (Jonason & Webster, 2010) is a brief measure of the Dark Triad traits, composed of the subscales of Machiavellianism (e.g. ‘I have used deceit or lied to get my way.’, 4 items), psychopathy (e.g. ‘I tend to lack remorse.’, 4 items) and narcissism (e.g. ‘I tend to want others to admire me.’, 4 items). Items are rated from 1 (*strongly disagree*) to 5 (*strongly agree*), and all items were averaged to produce a total dark triad score (*α* = .82).

#### Perpetration of dehumanisation

The short perpetration version of the Dehumanisation in Romantic Relationships Scale (Pizzirani et al., 2019) was used to assess dehumanisation perpetration. The measure consists of 6 items such as ‘generally I treat my partner as if they embarrass me’. Items are rated on a 7‐point scale ranging from 1 (*never*) to 7 (*always*) and averaged with higher scores indicative of greater dehumaniation perpetration (*α* = .76).

### Inhibitors/(dis)inhibitors (measured at baseline)

#### Constructive communication

A 3‐item version of the compromise subscale of the Romantic Partner Conflict Scale (Zacchilli et al., 2009) was used to assess constructive communication. Items are rated on a 5‐point scale from 0 (*strongly disagree*) to 4 (*strongly agree*). Scale items were averaged such that higher scores were indicative of greater constructive communication (*α* = .95).

#### Relationship quality

The Perceived Relationship Quality Components (PRQC) scale (Fletcher et al., 2000) was used to assess relationship quality. The measures consist of 18 items that assess six facets of relationship quality (3 items each). These domains are satisfaction, commitment, trust, intimacy, love and passion. Items are rated on a 7‐point scale ranging from 1 (*not at all*) to 7 (*extremely*), and items are averaged to form a total score for relationship quality, with higher scores indicating greater relationship quality (*α* = .91).

#### Partner support

Perceived partner support was assessed using four items developed for the study based on established measures of partner support (e.g. Cutrona & Russell, 2017; Feeney & Thrush, 2010). Two items measured the perception of esteem support (i.e. encouragement, affirmation of skills and abilities) and two items measured comfort support (i.e. validation, comfort and understanding) provided by one's partner. All items were rated on a 7‐point scale from 1 (*strongly disagree*) to 7 (*strongly agree*). Items were averaged such that higher scores indicate greater perceived partner support (*α* = .87).

#### Alcohol use

Alcohol use was assessed using the short version of Alcohol Use Disorders Identification Test—Consumption (AUDIT‐C, Bradley et al., 2007) which consists of three items measured on a 0–4 scale with differing anchors pertaining to drinking frequency. Scores range from 0 to 12, with scores of 4 and above for women and 3 and above for men indicating that the frequency of alcohol consumption is affecting a person's physical health and safety (*α* = .82).

#### Drug use

Drug use was assessed using the short version of Drug Use Disorders Identification Test—Consumption (DUDIT‐C, Berman et al., 2015) which consists of four items measured on a 0–4 scale with differing anchors pertaining to drug use frequency. Scores range from 0 to 16, with scores of 5 and above indicative of likely drug dependence (Pape et al., 2022; *α* = .81).

#### Non‐pandemic stressors and COVID‐19 stressors

A modified version of the Life Events/Hassles and Stress Scale (Holmes & Rahe, 1967) which contains 15 items assessing the prevalence of particular life events (e.g. ‘the death of someone close’, ‘change in your job/employment’) was answered dichotomously (i.e. ‘yes’ or ‘no’). Life events responded to with ‘yes’ were followed up with the dichotomous item ‘Was this event a result of COVID‐19?’. Finally, for each life event that occurred, the measure asked, ‘How much stress did you experience?’ rated on a 10‐point scale ranging from 1 (*very little stress*) to 10 (*very stressful*). *To determine an index for the stress of life events not due to COVID‐19*, a total score was obtained by summing the stress items for life events that were indicated as not being due to COVID‐19. Higher scores indicated greater stress from non‐COVID life events (*α* = .85).

### Intimate partner violence (measured at all 10 waves)

The shortened version of the Abuse Within Intimate Relationships Scale (AIRS; Borjesson et al., 2003) was used to assess how often acts of physical and psychological abuse occurred in the relationship. The measure included 14 items consisting of five subscales: overt physical violence (2 items), restrictive violence (2 items), emotional abuse (4 items), deception (3 items) and verbal abuse (3 items). All items were rated on a 3‐point scale ranging from 0 (*never*) to 2 (*twice or more*). All items were dichotomised such that scores of 1 or more were rescored as 1. This facilitated the derivation of a frequency count with regard to different forms of IPV. *Physical IPV* was assessed by summing the items (dichotomised) from the overt physical violence and restrictive violence subscales (*α* = .86). *Psychological IPV* was assessed by summing items (dichotomised) from the emotional abuse, deception and verbal abuse subscales (*α* = .88). Higher scores reflect higher acts of physical and psychological IPV perpetration, respectively.

### Data analysis

Analyses were conducted using Mplus 8.10 (Muthén & Muthén, 2023) and JASP 0.16 (JASP Team, 2022). Full information maximum likelihood (FIML) estimation was used in handling missingness. In terms of missing data, all participants completed at least two assessment waves, and 64.5% of the sample had completed five or more (50%+) assessment waves. The analyses were conducted in three stages. First, unconditional latent growth curve models (LGCM) for linear, quadratic, cubic and quartic growth trajectories were estimated for psychological and physical abuse separately across the ten time points for the entire sample. The estimation of unconditional LGCMs on the entire sample prior to the inclusion of any predictor variables is in line with guidelines for taking a step‐wise approach to LGCM testing and model building (e.g. Curran et al., 2010; Duncan & Duncan, 2009). All models included an intercept to reflect baseline levels of physical and psychological IPV, and a slope reflective of the trajectory of change. Chi‐square difference tests (Δχ^2^) of model fit were performed, and key goodness‐of‐fit indices including the Akaike Information Criterion (AIC) and Bayesian Information Criterion (BIC and sample size‐adjusted BIC) were examined to determine the trajectory of best fit for physical and psychological IPV.

Second, exploratory latent profile analysis (LPA) was conducted on baseline assessments of instigating, impelling and (dis)inhibiting factors. Given that all variables constituting instigating, impelling and (dis)inhibiting factors were measured using scales that varied in their scale ranges, all measures were standardised by converting them into a proportion of the maximum possible score (POMP) (Cohen et al., 1999) such that each measure ranged from 0 to 100. LPAs were estimated with increasing profile numbers (from 2 to 4), using a profile‐invariant diagonal variance–covariance matrix. The optimal latent profile solution was based on several goodness‐of‐fit indices, including Vuong–Lo–Mendell–Rubin (VLMR) and Lo–Mendell–Rubin (LMR) likelihood ratio test *p*‐values, AIC and BIC values as well as entropy values, profile assignment probability (>0.80; Rost, 2006), parsimony and the interpretability of latent profiles.

Third, conditional LGCMs were estimated separately for physical and psychological IPV. Latent profile membership and participants' state of residence (Victoria versus not living in Victoria) were predictors.

Apriori power analysis for unconditional and conditional LGCMs revealed that the minimum sample size required was 245 to detect the difference between an acceptable (RMSEA ~ 0.80) and mis‐specified (RMSEA ~ 0.11) model with a minimum of 35 degrees of freedom (*α* = .05) and a model power of 0.80.

## RESULTS

The results are presented in three parts. First, the unconditional LGCMs for physical and psychological IPV perpetration are reported. Second, the results of the LPA are reported. Finally, the conditional LGCMs are reported.

### Unconditional LGCMs

**FIGURE 1:**
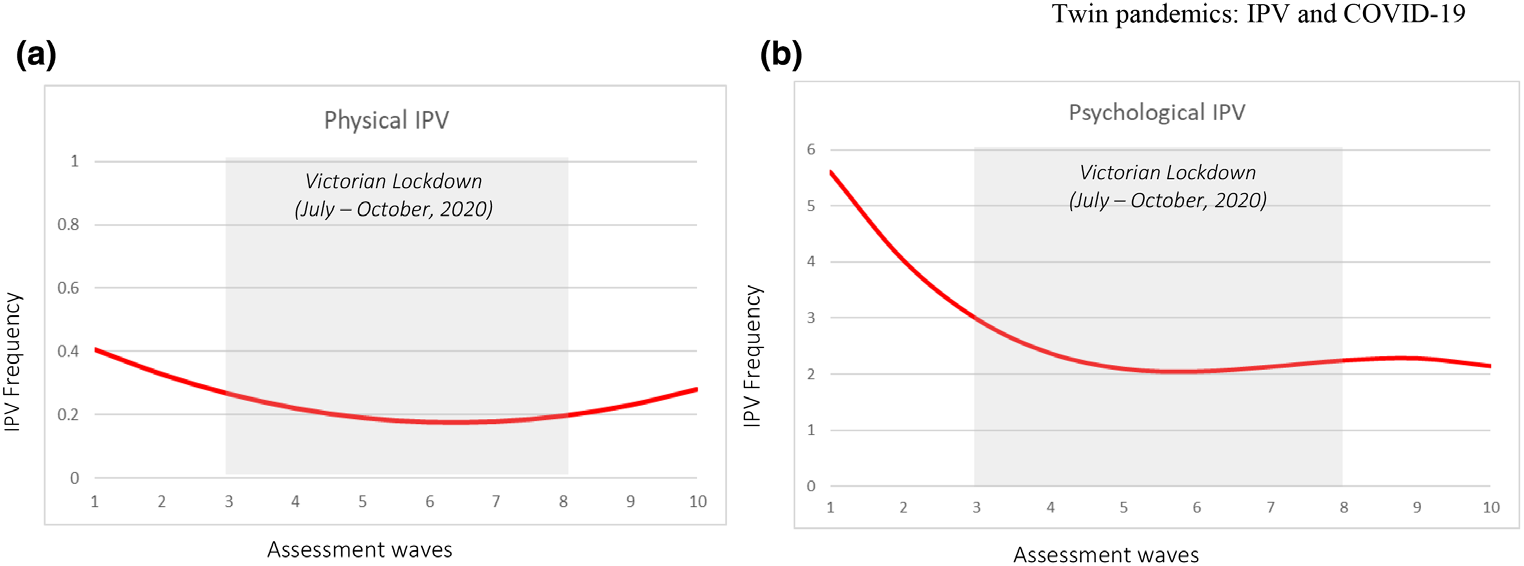
The latent growth curve trajectory of physical and psychological IPV perpetration across the 10 waves of assessment.

Table 1 presents the fit indices for the growth trajectories (i.e. linear, quadratic, cubic and quartic) for physical and psychological IPV. Chi‐square difference tests of model fit demonstrated that a quadratic trajectory fit the data best for physical IPV, but that a cubic trajectory fit the data best for psychological IPV (see Table 1). The estimated quadratic trajectory for physical IPV and the cubic trajectory for psychological IPV are shown in Figure 1a,b for the full sample. As shown in Figure 1a, physical IPV showed a decline prior to the strict lockdown for Victoria, with physical IPV continuing to reduce (but at a slower rate) for the whole sample during the strict Victorian lockdown period, followed by a slight upward increase following the easing of lockdown restrictions. Psychological IPV (see Figure 1b) showed a steady decline for the whole sample prior to and during the early stages of the Victorian lockdown, then plateaued before showing a slight increase mid‐way through the Victorian lockdown (when there was an easing of restrictions) and then another slight decrease.

**TABLE 1:** Identifying the average physical and psychological IPV perpetration trajectories: latent growth curve modelling fit estimates.

Model	X^2^	*df*	CFI	TLI	RMSEA	SRMR	Log‐like	AIC	BIC	SS‐BIC
Physical IPV										
Linear	284.96	42	0.88	0.871	0.098	0.135	−3203.56	6453.11	6554.55	6481.53
Quadratic	218.64	39	0.911	0.898	0.087	0.137	−3170.40	6392.79	6507.46	6424.91
Cubic	Estimation fail									
Psychological IPV										
Linear	281.011	42	0.889	0.881	0.097	0.088	−7901.73	15,849.47	15,950.9	15,877.88
Quadratic	223.115	39	0.914	0.901	0.088	0.083	−7872.79	15,797.57	15,912.24	15,829.69
Cubic	164.797	35	0.940	0.922	0.078	0.067	−7843.63	15,747.26	15,879.56	15,784.32
Quartic	Estimation fail									

### Latent profile analysis (LPA)

**FIGURE 2:**
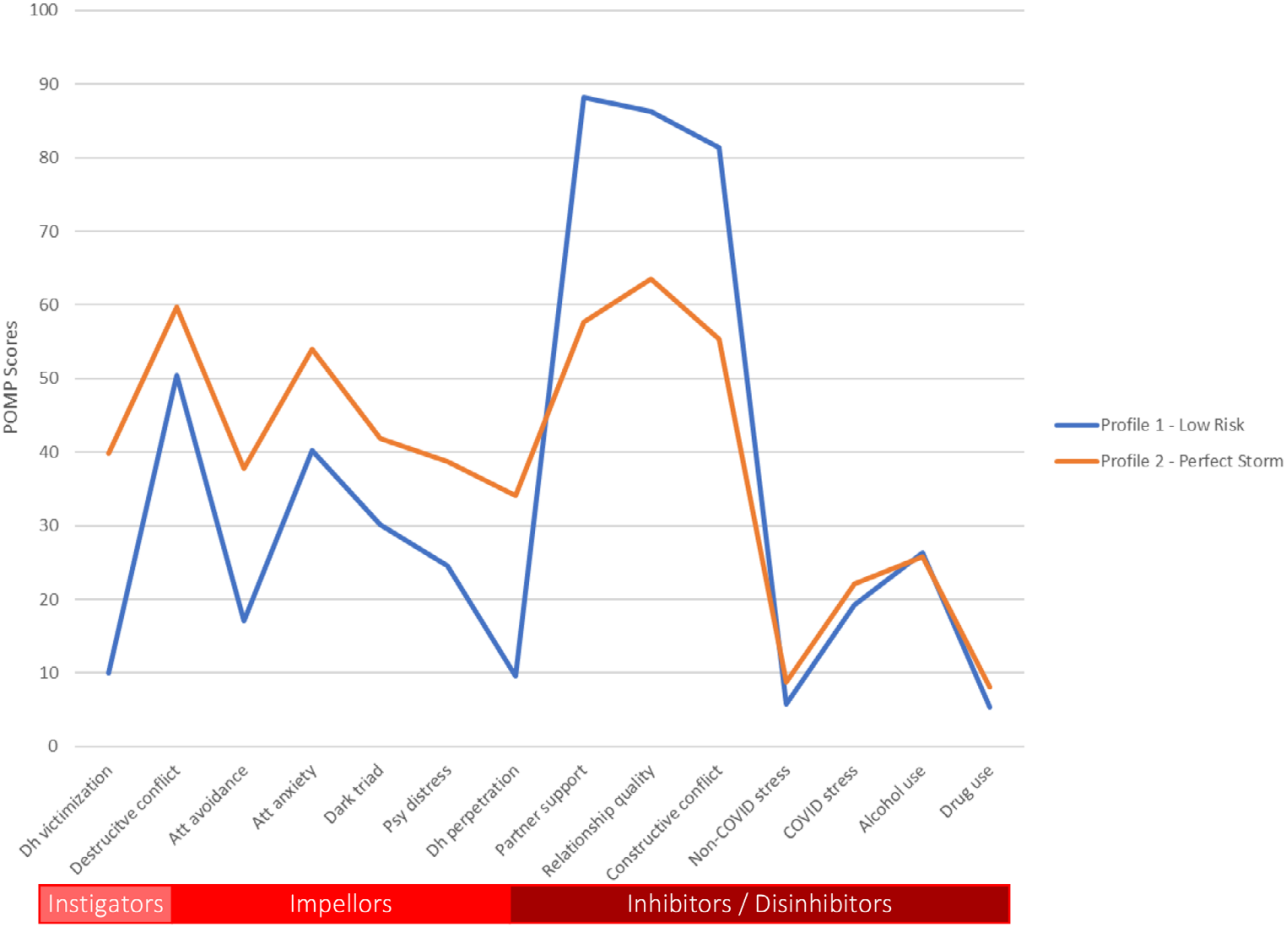
Mean proportion of the maximum possible scores for the constructs associated with the impellor, instigator and (dis)inhibitor factors for the low‐risk and perfect storm latent profiles. The impellor factor includes the constructs: att (attachment) avoidance and anxiety, dark triad, psychological distress and deh (dehumanisation) perpetration; the instigator factor includes the constructs: deh (dehumanisation) victimisation and destructive conflict patterns; the (dis)inhibitor factor includes the constructs: stress (COVID, pre‐COVID/non‐pandemic stress), constructive conflict communication, partner support, relationship quality, alcohol use and drug use.

Table 2 presents the fit statistics for the LPA, which tested 2–4 LPA solutions. As shown in Table 3, a 2‐profile solution best represented the sample. Although the AIC and BIC values for the 3‐ and 4‐profile solutions were lower than the 2‐profile solution, the 4‐profile solution produced one group that had a cell size under 4% and the 2‐profile solution had the highest entropy. Furthermore, the LMR and VLMR *p*‐values indicated that a 2‐profile solution provided a better fit than a 1‐profile solution, and the 3‐ and 4‐profile solutions did not provide a significantly better fit. Thus, high profile assignment probability was obtained for the 2‐profile solution (Profile 1 [.99], Profile 2 [.96]). Profile 1 (P1) consisted of 463 participants (76%) and Profile 2 (P2) consisted of 145 participants (24%). As shown in Figure 2, the profile of mean scores for the two profiles revealed that P2 was higher than P1 on instigators and impellors, and lower on inhibitors (except for three [dis]inhibitors—alcohol and drug use and stress—which were similarly low for both profiles). We have labelled P2 the ‘perfect storm’ profile and P1 the ‘low‐risk’ profile.

**TABLE 2:** Identifying the profiles for the instigating, impelling and (Dis)inhibiting factors: latent profile solution indices.

Profiles (*k*)	Log likelihood	AIC	BIC	VLMR	LMR	Entropy	Profile	*N* (full sample)	%
				*p*‐value	*p*‐value				
2	−32,756.76	65,593.51	65,769.92	0	0	0.931	1	464	76
							2	144	27
3	−32,588.62	65,285.24	65,523.39	.08	.09	0.918	1	343	56
							2	124	21
							3	141	23
4	−32,455.59	65,047.17	65,347.07	.23	.22	0.906	1	24	4
							2	129	21
							3	135	22
							4	320	53

**TABLE 3:** Conditional LGCM estimates for physical and psychological IPV.

Predictor	Growth parameter	Physical IPV				Psychological IPV			
		95% confidence interval				95% confidence interval			
		Estimate	SE	Lower	Upper	Estimate	SE	Lower	Upper
Latent profile group (0 = low risk, 1 = perfect storm)	Intercept	0.51***	0.11	0.30	0.73	3.448***	0.42	2.64	4.26
	Linear	−0.12**	0.041	−0.21	−0.03	−0.66*	0.34	−1.32	−0.0004
	Quadratic	0.008*	0.004	−15.41	−0.02	0.10	0.08	−0.07	0.26
	Cubic	–	–	–	–	−0.01	0.01	−0.02	0.01
State of residence (0 = not Victoria, 1 = Victoria)	Intercept	0.17	0.10	−0.02	0.36	−0.11	0.37	−0.82	0.61
	Linear	−0.04	0.04	−0.11	0.04	0.10	0.30	−0.48	0.68
	Quadratic	0.002	0.004	−0.01	0.01	−0.07	0.07	−0.21	0.08
	Cubic	–	–	–	–	0.006	0.005	−0.005	0.02

* *p* < .05.

** *p* < .01.

*** *p* < .001.

### Conditional LGCMs

To determine whether the two latent profile groups and lockdown status were significant predictors of IPV trajectories, we next conducted two conditional LGCMs—one for physical IPV (based on the quadratic trajectory) and one for psychological IPV (based on the cubic trajectory). Both predictors were included as dichotomous variables (latent profile: 0 = low‐risk profile, 1 = perfect storm profile; state of residence: 0 = not Victoria, 1 = Victoria). As shown in Table 3, the latent profile group variable was the only significant predictor of the quadratic and cubic trajectories of physical and psychological IPV, respectively. In terms of the specific growth parameters, the latent profile group variable significantly predicted the intercept and linear estimates for both types of IPV, and it also was a significant predictor of the quadratic estimate for physical IPV.^3^

**FIGURE 3:**
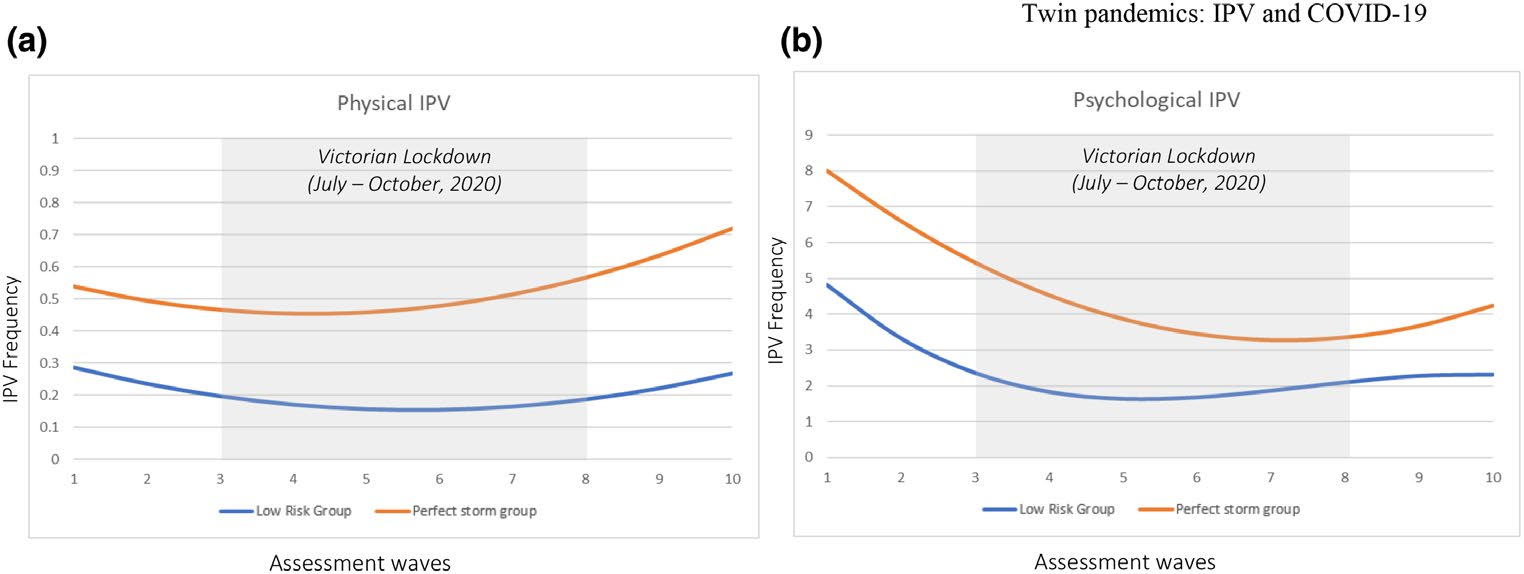
Latent growth curve trajectories for physical and psychological intimate partner violence (IPV) for the low‐risk and perfect storm profile groups.

Figure 3 illustrates the trajectories of the two profile groups for both types of IPV. For physical IPV (see Figure 3a), both groups showed a quadratic trajectory. However, the perfect storm group had a higher mean level of IPV at baseline, with the perpetration of physical IPV remaining higher across all 10 waves compared with the low‐risk group. Furthermore, the perfect storm profile group had a steeper acceleration in physical IPV across a 10‐week period from waves 6 through 10, with lockdown restrictions for Victoria easing at wave 8. For psychological IPV (see Figure 3b), the perfect storm group had a higher mean level of IPV at baseline and perpetration remained higher compared with the low‐risk profile group across all 10 assessment waves. In addition, the perfect storm group showed an upward acceleration in psychological IPV over waves 8–10 (during the lifting of the Victorian lockdown), whereas the low‐risk group had a plateauing in psychological IPV across the same period.

## DISCUSSION

The findings of this study directly address whether the social restrictions brought on by the COVID‐19 pandemic heightened the perpetration of IPV within the general community. Our findings suggested that lockdown status (Victorian citizens subjected to strict lockdown compared to non‐Victorian citizens who were not) was not directly associated with the trajectories of physical or psychological IPV over a five‐month period. Our findings bring into question the “twin pandemics” notion put forth by those who suggest that government‐mandated COVID‐19 social restrictions were directly associated with significant increases in rates of IPV across the globe (Evans et al., 2020). Indeed, our findings align with recent evidence to suggest that COVID‐19 social restrictions were not directly associated with any discernible change in patterns and rates of IPV within Australia (Miller et al., 2023).

Furthermore, our findings suggest that people's profiles of instigating, impelling and inhibiting factors were directly predictive of the trajectories of IPV perpetration. Our latent profile analysis revealed two profiles based on the I^3^ model. The first was a ‘perfect storm profile’ indexed by higher instigators (i.e. dehumanisation victimisation and destructive conflict patterns), higher impellors (i.e. attachment insecurity, dark triad traits, psychological distress and dehumanisation perpetration tendencies) and lower inhibitors (i.e. partner support, relationship quality, constructive conflict patterns). The second, was a low‐risk profile that reflected the inverse of the perfect storm profile. However, the two profiles did not differ on (dis)inhibiting factors, namely, life stress (COVID‐19 and non‐COVID‐19 stress) and substance use (alcohol and other drugs).

In line with the I^3^ model, individuals characterised by a perfect storm profile demonstrated elevated perpetration of both physical and psychological IPV over time relative to individuals who were lower on instigators and impellors and higher on inhibitors (i.e. the low‐risk profile). These latent profiles were also predictive of different rates of acceleration and deceleration in the perpetration of IPV across the pandemic. Specifically, individuals who had a perfect storm profile (compared to those who had a low‐risk profile) had steeper acceleration in physical IPV mid‐way through the study (coinciding with the tightening of Victorian lockdown restrictions), with the rate of acceleration increasing for a month following the easing of Victorian lockdown restrictions. Furthermore, the perfect storm group showed an acceleration of psychological IPV (whereas the low‐risk group showed a plateauing) near the end of the study, during the time when Victoria ended its lockdown restrictions.

These findings are significant for several reasons. First, the non‐linear trajectories of physical and psychological IPV that we identified highlight the need to monitor and repeatedly assess IPV during health crises. Indeed, repeated assessments can provide better insights into the thresholds at which IPV is likely to increase and decrease across time. In the digital age, this can be accomplished on a universal level by using brief, online assessments of instigating, impelling and inhibiting factors to identify those at greatest risk of IPV escalation. In the present study, there was a reduction in both physical and psychological IPV for both the perfect storm group and the low‐risk group across the five‐month period, irrespective of lockdown status. However, for the perfect storm group, IPV perpetration accelerated upwards when the entire country was no longer subject to social restrictions. In other words, IPV increased at a faster rate when no lockdown restrictions were in place anywhere in Australia than when restrictions were in place within the state of Victoria (irrespective of participants' state of residence).

Second, the findings reveal that IPV rates can be explained by the confluence of certain individual and contextual factors theorised to contribute to the perpetration of IPV, and that it is critical to adopt an integrative approach to understanding IPV across the COVID‐19 pandemic. Our findings reveal that assessing critical instigators, impellors and inhibitors allows one to identify individuals who are at greater risk of perpetrating IPV. These insights can help governments and policymakers identify those individuals who are most likely to aggress against their romantic partners.

Third, our latent profile analysis revealed that the most marked differences between the perfect storm group and the low‐risk group involved: (a) the instigator of being a victim of their partner's dehumanising behaviour, (b) certain impellors (especially attachment insecurity and the perpetration of dehumanisation) and (c) inhibitors (i.e. the provision of partner support, higher relationship quality and more constructive conflict behaviours). These variables all reflect relationship‐specific factors that are either modifiable (as in the case of support processes, relationship quality and the enactment of negative relationship behaviours such as partner dehumanisation) or individual difference factors (such as attachment insecurity) that can be directly addressed as part of evidence‐based relationship education or therapy programmes.

These findings are especially significant when put into the context of recent evidence that supports the role of targeting relationship factors in addressing relationship aggression. For instance, a recent meta‐analysis of relationship education programmes that target developing relationship skills (e.g. communication patterns, conflict resolution and support processes) was found to reduce relationship aggression, especially among individuals who reported moderate‐to‐severe IPV prior to programme commencement (Karantzas, Curtis, et al., 2023). Furthermore, several evidence‐based couple therapies such as Emotionally Focused Couples Therapy (Johnson, 2019) and Integrative Behavioural Couples Therapy (Christensen et al., 2020) target underlying vulnerability factors and impellors such as attachment insecurity, psychological distress and other aspects of personality when treating relationship dysfunction, including aggression.

Within the context of COVID‐19 and social restrictions, the delivery of relationship education or couple therapy requires online technologies and the implementation of digital innovations. Governments and service providers can, therefore, leverage existing programmes that provide self‐directed online relationship education and counselling. Two examples are the ePREP and the Our Relationship programmes, both of which are adaptations of established programmes (Prevention and Relationship Enhancement Programme [PREP] and Integrative Behavioural Couple Therapy; Doss et al., 2020). Further, research has confirmed that both online programmes enhance relationship functioning and reduce relationship aggression (Doss et al., 2020).

### Limitations and future research

Although our study has several strengths and reveals novel insights into IPV during the COVID‐19 pandemic, it has some limitations. Neither of the latent profiles in our study showed high COVID‐19 or pre‐pandemic stress, nor did either profile demonstrate problematic substance use. Thus, future research should investigate IPV using integrative frameworks within sub‐populations that focus more on stress and substance misuse given that these instigating and (dis)inhibiting factors can be important drivers of IPV (Eckhardt et al., 2019). Furthermore, despite efforts to obtain a socio‐economic and culturally diverse, gender‐balanced sample, our sample primarily consisted of white/European participants, approximately 60% women, and findings are limited to the Australian context. Although our supplementary analyses (see footnote 3 and Tables S1–S10) did not suggest that gender (i.e. cisnormative men and women) and cultural background (i.e. White/European vs. non‐White/European) moderated IPV perpetration trajectories, future research should examine the generalisability of our findings in more culturally and gender‐diverse groups, as well as other nations. We also acknowledge that the lack of gender and cultural differences may be associated with our choice of IPV measurement which was based on summing the presence of acts related to physical and psychological IPV rather than an assessment of violence severity. Therefore, examining severity alongside the presence/absence of particular IPV behaviours would be an important future extension of the current work.

Also, although our findings evidenced no direct association between social restrictions and IPV perpetration, we cannot discount that social restrictions may have yielded an indirect effect on IPV through the possible elevation of I^3^ variables that can evidence change during periods of stress and strain. These could include upward shifts in psychological distress, dehumanisation, substance misuse and destructive conflict as well as reductions in adaptive relational processes. Thus, future research could focus on testing possible indirect pathways through which social restrictions may affect IPV perpetration. Finally, IPV occurs within the context of dyadic relationships, so future work should recruit couples to model how the impelling, instigating and inhibiting factors of both partners contribute to each partner's IPV trajectory, including the covariation between the partners' IPV trajectories.

## CONCLUSION

In summary, our findings suggest that the perpetration of IPV is not necessarily tethered to social restrictions (i.e. lockdown orders) enforced during COVID‐19. Rather, our findings provide important insights into identifying: (a) the individuals, (b) the modifiable factors and (c) the points in time at which interventions might be most successful in reducing the perpetration of IPV. These insights, in other words, are likely to extend to risk profiling of IPV perpetration as it relates to health‐related disasters and crises beyond the COVID‐19 pandemic.

## AUTHOR CONTRIBUTIONS

**Gery C. Karantzas:** Conceptualization; investigation; writing – original draft; methodology; writing – review and editing; formal analysis; project administration; supervision; resources. **Daniel A. Romano:** Methodology; formal analysis; data curation; project administration; writing – review and editing. **Susan Chesterman:** Methodology; writing – review and editing; data curation; project administration. **Emma M. Marshall:** Writing – review and editing. **Laura Knox:** Methodology; writing – review and editing; project administration; data curation. **Ellie R. Mullins:** Methodology; project administration; writing ‐ review and editing; **Nicholas Lawless:** Methodology; writing – review and editing. **Elizabeth Ferguson:** Methodology; project administration; writing – review and editing. **Peter G. Miller:** Resources; writing – review and editing; conceptualization. **Christopher I. Eckhardt:** Conceptualization; writing – review and editing. **Pam Pilkington:** Writing – review and editing. **Anshu Patel:** Writing – review and editing. **Jeffry A. Simpson:** Conceptualization; writing – review and editing.

## CONFLICT OF INTEREST STATEMENT

The authors have no conflicts of interest to declare.

## Supporting information


Table S1.

## Data Availability

The data that support the findings of this study are available on request from the corresponding author. The data are not publicly available due to privacy or ethical restrictions.

## Footnotes

^1^
As part of state government‐mandated lockdown orders, Victorians were required to always remain in their homes except for one member per household allowed to leave their residence (once per day) to purchase essential shopping and goods. Citizens were allowed to exercise for a maximum of 1 h per day within a 5‐km radius of one's home, the wearing of masks was mandatory both indoors and outdoors for those aged 12 years and above, and a night‐time curfew was enforced between the hours of 8 PM and 5 AM. Early childhood education, schools and higher education institutions were closed, and non‐essential workers were required to work from home. At the time, the Victorian lockdown would become one of the strictest and longest in the world, spanning 4 months from June to October 2020.

^2^
Due to institutional ethics requirements, data are not available on an open repository. Data access can be requested by submitting a formal proposal to the corresponding author.

^3^
We conducted two sets of supplementary analyses. The first set of these supplementary analyses was to cross‐validate our primary findings (see Tables S1–S10). We ran LGCMs separately for: (1) Victorian and non‐Victorian participants, and (2) latent profile groups. We conducted these supplementary analyses to confirm if the best fitting trajectories evidenced for psychological and physical IPV matched the findings for the overall sample. We found that for both the Victorian and non‐Victorian participants groups that a quadratic trajectory and cubic trajectory provided significantly better fit to the data than all other trajectories for physical and psychological IPV, respectively. The same findings were evidenced when assessing the best‐fitting trajectories for the two latent profile groups. That is, for both the low risk and perfect storm groups, a quadratic trajectory best fit the data for physical IPV and a cubic trajectory best fit the data for psychological IPV. The second set of supplementary analyses focused on determining whether gender and cultural background moderated the best fitting trajectories for physical and psychological IPV. Although not a focus of this research study, we conducted these analyses given extant research into gender‐based violence (e.g. Tadesse et al., 2024) and cultural determinants of violence (e.g. Mallory et al., 2016). First, moderated LGCMs were conducted testing whether gender (i.e. cisnormative men vs. women) moderated the trajectories for physical and psychological IPV. As shown in the Tables S1–S10, gender was not found to moderate the trajectories for both forms of IPV. Second, moderated LGCMs were conducted testing whether cultural background (i.e. participants who identified as white/European vs. those who identified as non‐white/non‐European) moderated the trajectories of physical and psychological IPV. Again, cultural background was not found to moderate the trajectories for both forms of IPV.
