# Supplementary material for: The ‘twin pandemics’? modelling and predicting the trajectories of IPV perpetration during the COVID‐19 pandemic in Australia
Source: Br J Soc Psychol. 2025 Jul 23;64(3):e12911. doi: 10.1111/bjso.12911 (PMC12285568; doi:10.1111/bjso.12911)
Supplement: Supplementary file 1 — Table S1. [file BJSO-64-0-s001.docx]

Supplementary Material

Table of Contents

Table S1. …………………………………………………………………………..2

Table S2. …………………………………………………………………………..3

Table S3. …………………………………………………………………………..4

Table S4. …………………………………………………………………………..5

Table S5. …………………………………………………………………………..6

Table S6. …………………………………………………………………………..7

Table S7. …………………………………………………………………………..8

Table S8. …………………………………………………………………………..9

Table S9. ………………………………………………………………………….10

Table S10. …………………………………………………………………………..11

**Table S1**

**Low Risk Profile (*n* = 467) model trajectory fit measures and comparison – Psychological IPV**

| **Model** | **χ²** | **df** | **CFI** | **TLI** | **AIC** | **BIC** |
| --- | --- | --- | --- | --- | --- | --- |
| Linear | 274.15 | 42 | 0.873 | 0.864 | 11969.80 | 12065.17 |
| Quadratic | 213.673 | 39 | 0.905 | 0.890 | 11915.33 | 12023.13 |
| Cubic | 178.645 | 35 | 0.922 | 0.900 | 11888.30 | 12012.69 |

Model difference tests

Linear model vs Quadratic model: Δχ^2^(3) = 60.477, *p* < .0001; ΔTLI = .027; ΔAIC = 54.47; ΔBIC = 42.04.

Quadratic model vs Cubic model: Δχ^2^(4) = 35.028, *p* < .0001; ΔTLI = .010; ΔAIC = 27.03; ΔBIC = 10.44.

**Table S2**

**Perfect Storm Profile (*n* = 141) model trajectory fit measures and comparison – Psychological IPV**

| **Model** | **χ²** | **df** | **CFI** | **TLI** | **AIC** | **BIC** |
| --- | --- | --- | --- | --- | --- | --- |
| Linear | 107.521 | 42 | 0.833 | 0.821 | 3663.76 | 3731.58 |
| Quadratic | 93.050 | 39 | 0.862 | 0.841 | 3655.29 | 3731.95 |
| Cubic | 62.718 | 35 | 0.929 | 0.909 | 3632.95 | 3721.42 |

Model difference tests

Linear model vs Quadratic model: Δχ^2^(3) = 14.47, p < .0001; ΔTLI = .020; ΔAIC = 8.47; ΔBIC = -.037.

Quadratic model vs Cubic model: Δχ^2^(4) = 30.33, p < .0001; ΔTLI = .068; ΔAIC = 22.34; ΔBIC = 10.53.

**Table S3**

**Victorian participants (*n* = 217) model trajectory fit measures and comparison – Psychological IPV**

| **Model** | **χ²** | **df** | **CFI** | **TLI** | **AIC** | **BIC** |
| --- | --- | --- | --- | --- | --- | --- |
| Linear | 146.887 | 42 | 0.848 | 0.838 | 5229.25 | 5306.99 |
| Quadratic | 142.145 | 39 | 0.851 | 0.828 | 5230.51 | 5318.39 |
| Cubic | 105.924 | 31 | 0.892 | 0.843 | 5210.29 | 5325.20 |

Model difference tests

Linear model vs Quadratic model: Δχ^2^(3) = 4.74, p > .05; ΔTLI = .010; ΔAIC = -1.26; ΔBIC = -11.40.

Quadratic model vs Cubic model: Δχ^2^(8) = 36.22, p < .0001; ΔTLI = .015; ΔAIC = 20.22; ΔBIC = -6.91

**Table S4**

**Non-Victorian participants (*n* = 391) model trajectory fit measures and comparison – Psychological IPV**

| **Model** | **χ²** | **df** | **CFI** | **TLI** | **AIC** | **BIC** |
| --- | --- | --- | --- | --- | --- | --- |
| Linear | 259.96 | 42 | 0.855 | 0.845 | 10527.56 | 10618.84 |
| Quadratic | 199.95 | 39 | 0.893 | 0.877 | 10473.55 | 10576.73 |
| Cubic | 164.09 | 35 | 0.914 | 0.890 | 10445.69 | 10564.75 |

Model difference tests

Linear model vs Quadratic model: Δχ^2^(3) = 60.01, p < .0001; ΔTLI = .032; ΔAIC = 54.01; ΔBIC = 42.11.

Quadratic model vs Cubic model: Δχ^2^(8) = 35.86, p < .0001; ΔTLI = .021; ΔAIC = 27.86; ΔBIC = 11.98

**Table S5**

**Low Risk Profile (*n* = 467) model trajectory fit measures and comparison – Physical IPV**

| **Model** | **χ²** | **df** | **CFI** | **TLI** | **AIC** | **BIC** |
| --- | --- | --- | --- | --- | --- | --- |
| Linear | 280.532 | 42 | 0.881 | 0.873 | 4382.987 | 4478.352 |
| Quadratic | 247.833 | 39 | 0.896 | 0.889 | 4356.288 | 4464.092 |

Model difference tests

Linear model vs Quadratic model: Δχ^2^(3) = 32.70, p < .0001; ΔTLI = .015; ΔAIC = 26.70; ΔBIC = 14.26.

**Table S6**

**Perfect Storm Profile (*n* = 141) model trajectory fit measures and comparison – Physical IPV**

| **Model** | **χ²** | **df** | **CFI** | | **TLI** | | **AIC** | | **BIC** | |
| --- | --- | --- | --- | --- | --- | --- | --- | --- | --- | --- |
| Linear | 123.187 | 42 | 0.736 | 0.717 | | 3703.18 | | 3771.00 | |  |
| Quadratic | 79.054 | 39 | 0.870 | | 0.850 | | 3652.14 | | 3728.81 | |

Model difference tests

Linear model vs Quadratic model: Δχ^2^(3) = 44.13, p < .0001; ΔTLI = .133; ΔAIC = 51.04; ΔBIC = 42.19.

**Table S7**

**Non-Victorian participants (*n* = 391) model trajectory fit measures and comparison – Physical IPV**

| **Model** | **χ²** | **df** | **CFI** | **TLI** | **AIC** | **BIC** |
| --- | --- | --- | --- | --- | --- | --- |
| Linear | 217.762 | 42 | 0.871 | 0.862 | 4035.47 | 4126.75 |
| Quadratic | 195.502 | 39 | 0.885 | 0.867 | 4019.21 | 4122.40 |

Model difference tests

Linear model vs Quadratic model: Δχ^2^(3) = 22.26, p < .0001; ΔTLI = .005; ΔAIC = 16.26; ΔBIC = 4.35.

**Table S8.**

**Victorian participants (*n* = 217) model trajectory fit measures and comparison – Physical IPV**

| **Model** | **χ²** | **df** | **CFI** | **TLI** | **AIC** | **BIC** |
| --- | --- | --- | --- | --- | --- | --- |
| Linear | 180.810 | 42 | 0.748 | 0.730 | 8045.88 | 8132.33 |
| Quadratic | 103.025 | 39 | 0.884 | 0.866 | 7917.08 | 8014.81 |

Model difference tests

Linear model vs Quadratic model: Δχ^2^(3) = 77.79, p < .0001; ΔTLI = .136; ΔAIC = 128.80; ΔBIC = 117.52.

**Table S9.**

**Latent Growth Curve Model Moderated by Gender**

| **Component** | **Predictor** | **Estimate** | **Std. Error** | **z-value** | **p** | **95% CI**  **Lower** | **95% CI**  **Upper** | **Std. Est.** |
| --- | --- | --- | --- | --- | --- | --- | --- | --- |
| **Physical IPV** |  |  |  |  |  |  |  |  |
| Intercept | Gender | 0.052 | 0.098 | 0.531 | 0.596 | -0.140 | 0.245 | 0.055 |
| Linear slope | Gender | 0.005 | 0.039 | 0.128 | 0.898 | -0.071 | 0.081 | 0.017 |
| Quadratic slope | Gender | -0.002 | 0.004 | -0.700 | 0.484 | -0.009 | 0.004 | -0.111 |
| **Psychological IPV** |  |  |  |  |  |  |  |  |
| Intercept | Gender | 0.028 | 0.384 | 0.072 | 0.943 | -0.724 | 0.779 | 0.009 |
| Linear slope | Gender | -0.236 | 0.299 | -0.79 | 0.430 | -0.822 | 0.35 | -0.143 |
| Quadratic slope | Gender | 0.096 | 0.074 | 1.301 | 0.193 | -0.049 | 0.241 | 0.282 |
| Cubic slope | Gender | -0.008 | 0.005 | -1.567 | 0.117 | -0.018 | 0.002 | -0.453 |
| *Note.* Gender 1 = Male, 2 = Female | | |  |  |  |  |  |  |

**Table S10.**

**Latent Growth Curve Model Moderated by Cultural Background**

| **Component** | **Predictor** | **Estimate** | **Std. Error** | **z-value** | **p** | **Lower** | | **Upper** | **Std. Est.** |
| --- | --- | --- | --- | --- | --- | --- | --- | --- | --- |
| **Physical IPV** |  |  |  |  |  |  | |  |  |
| Intercept | Cultural Background | 0.046 | 0.113 | 0.406 | 0.685 | -0.175 | | 0.267 | 0.048 |
| Linear slope | Cultural Background | 0.003 | 0.045 | 0.077 | 0.939 | -0.085 | | 0.091 | 0.012 |
| Quadratic slope | Cultural Background | 0.001 | 0.004 | 0.155 | 0.877 | -0.007 | | 0.009 | 0.028 |
| **Psychological IPV** | | | | | | | | | |
| Intercept | Cultural Background | -0.794 | 0.441 | -1.802 | 0.072 | -1.658 | | 0.07 | -0.247 |
| Linear slope | Cultural Background | 0.393 | 0.345 | 1.138 | 0.255 | -0.284 | | 1.069 | 0.238 |
| Quadratic slope | Cultural Background | -0.047 | 0.086 | -0.546 | 0.585 | -0.215 | | 0.121 | -0.137 |
| Cubic slope | Cultural Background | 0.001 | 0.006 | 0.149 | 0.882 | -0.011 | | 0.013 | 0.05 |
| Cultural Background 0 = Non-white/non-European; 1 = White/European | | | | | | |  |  |  |
